# Direct Observations of Twin Formation Dynamics in Binary Semiconductors

**DOI:** 10.1021/acsnanoscienceau.1c00021

**Published:** 2021-11-04

**Authors:** Marcus Tornberg, Robin Sjökvist, Krishna Kumar, Christopher R. Andersen, Carina B. Maliakkal, Daniel Jacobsson, Kimberly A. Dick

**Affiliations:** † Centre for Analysis and Synthesis, Lund University, Box 118, 22100 Lund, Sweden; ‡ NanoLund, Lund University, 22100 Lund, Sweden; § National Centre for Nano Fabrication and Characterization, Technical University of Denmark, 2800 Kongens Lyngby, Denmark; ∥ National Center for High Resolution Electron Microscopy (nCHREM), Lund University, 22100 Lund, Sweden

**Keywords:** Environmental Transmission Electron Microscopy, Nanowires, GaAs, Stacking-Faults, Twinplane, Deterministic Crystal Growth, MOCVD, In Situ TEM

## Abstract

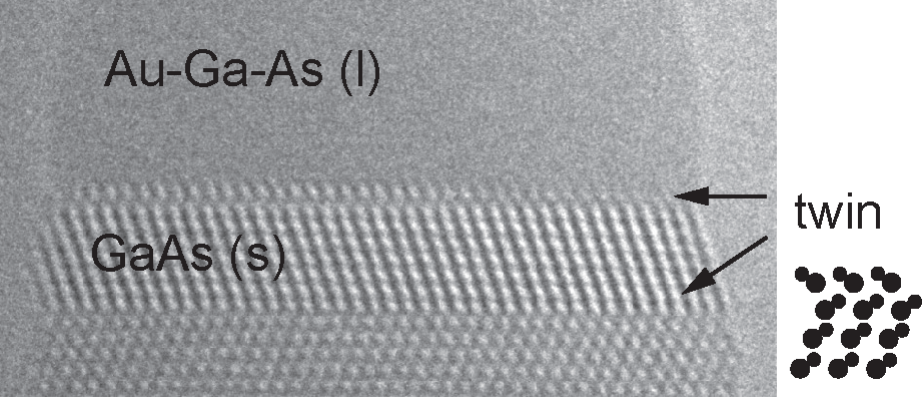

With the increased demand for controlled deterministic growth of III–V semiconductors at the nanoscale, the impact and interest of understanding defect formation and crystal structure switching becomes increasingly important. Vapor–liquid–solid (VLS) growth of semiconductor nanocrystals is an important mechanism for controlling and studying the formation of individual crystal layers and stacking defects. Using *in situ* studies, combining atomic resolution of transmission electron microscopy and controlled VLS crystal growth using metal organic chemical vapor deposition, we investigate the simplest achievable change in atomic layer stacking–single twinned layers formed in GaAs. Using Au-assisted GaAs nanowires of various diameters, we study the formation of individual layers with atomic resolution to reveal the growth difference in forming a twin defect. We determine that the formation of a twinned layer occurs significantly more slowly than that of a normal crystal layer. To understand this, we conduct thermodynamic modeling and determine that the propagation of a twin is limited by the energy cost of forming the twin interface. Finally, we determine that the slower propagation of twinned layers increases the probability of additional layers nucleating, such that multiple layers grow simultaneously. This observation challenges the current understanding that continuous uniform epitaxial growth, especially in the case of liquid-metal assisted nanowires, proceeds one single layer at a time and that its progression is limited by the nucleation rate.

## Introduction

Atomic-scale design of semiconductor nanocrystals is the ultimate challenge for novel devices and in particular new quantum technologies based on these materials. The formation of semiconductor nanocrystals from a liquid, termed the vapor–liquid–solid (VLS) mechanism, is a process considered to proceed almost exclusively one layer at a time.^1−3^ Theoretically, such layer-by-layer growth enables full control of each individually formed layer, making it extremely interesting for forming semiconductor crystals (“nanowires”) with precise control of structure and composition. This is especially critical when designing and growing heterostructures with atomically sharp interfaces between segments of different materials with specific lengths.^4−6^ The process also inherently allows for the intentional formation of twins and other stacking defects as desired, facilitating the growth of crystal phase heterostructures,^7,8^ which in turn enables the design of, for instance, quantum dots with strain-free interfaces.^9−11^ These changes require precise environmental control, excellent understanding of the growth process, along with outstanding engineering skills to achieve. However, to generalize and transfer the knowledge to other material systems, we need to understand the layer growth process at the nanoscale, in particular the formation of stacking defects. It is a grand task, which connects an understanding of how to control the purity of the crystal, with the design of crystals with intentionally introduced stacking defects.

Binary semiconductors such as GaAs are known to grow one bilayer (a layer consisting of Ga–As pairs) at a time^2,3^ and to crystallize in either a cubic (zinc blende) or hexagonal (wurtzite) structure when grown by the VLS mechanism along the ⟨111⟩ axis.^12,13^ Extensive control of crystal structure has been demonstrated by process engineering for GaAs and similar III–V semiconductor materials,^14,15^ down to precise crystal segment lengths of a few nanometers.^7,8^ While these postgrowth (“*ex situ*”) studies showcase excellent deterministic engineered control, this is achieved by changing the growth conditions to form the different crystal structures, making it difficult to compare and understand the formation processes of the different structures and individual stacking defects. As a result, the interpretation of the growth process using postgrowth analysis is based on averages over long segments rather than connecting conditions to specific layers. Moreover, surface energies and tensions are critical to determining the crystal structure but are dependent on the surrounding environment due to reformation or reconstructions,^16−18^ complicating direct comparison between the formation of different atomic arrangements (crystal phases).

These challenges can be avoided by studying the formation of crystal layers in real time within an environmental transmission electron microscope. Recent development of instrumentation for electron microscopy studies in a gaseous environment has enabled atomically resolved real-time investigations of crystal formation, transformation, and/or growth with atomic resolution.^3,19,20^ The ability to observe individual crystal layers forming in a steady state growth environment provides a way to investigate the formation of different atomic arrangements/stacking patterns. In contrast to current reports on defect formation with respect to the overall axial growth rate and growth regimes,^12,21−23^ it provides the possibility to separate the nucleation event from propagation of each layer and in turn enables detailed studies of growth dynamics for individual layers. So far, this type of *in situ* microscopy growth study has been key to investigate other aspects of the III–V semiconductor growth process such as the assisting droplet composition and growth dynamics,^20,24^ the evolution of a single layer at constant flow,^3^ and the droplet behavior during growth while altering the supply ratio of the growth species.^25,26^

In this paper, we study the dynamics of stacking defect formation during VLS GaAs nanowire growth with *in situ* transmission electron microscopy. To improve our understanding of crystal structure changes and occurrence and consequences of defect formation in binary semiconductors, we focus on the simplest case of defect and/or phase interface: the twin defect across the cubic (111) plane. In order to exclude the surface energy dependency on the environment from the analysis, we target formation of twin defects under conditions where they occur regularly within an otherwise zinc blende structure. This provides an internal reference using the cubic structured growth for each individual nanowire. For these conditions, we determine that while the time required to nucleate a new layer is similar for a layer that is twinned or in a normal configuration relative to the underlying crystal, the time required for that layer to propagate (grow) across the interface is much longer. Based on these observations, we develop a thermodynamic understanding of the growth using nucleation modeling and suggest that the energy cost of forming a twin interface limits the growth. In turn, this gives a high probability of multiple layers nucleating in a stack and propagating simultaneously. Understanding and properly considering these observations is essential for atomically precise control of crystal layers in semiconductor nanowires as well as for controllably forming more complex structures at this level.

## Method

To study the formation of individual crystal layers in VLS-grown GaAs nanowires, we grow the structures inside an environmental transmission electron microscope (ETEM; Hitachi HF3300S operated at 300 keV) which is directly connected to a metal organic chemical vapor deposition (MOCVD) system. The two systems are here interfaced via a side port to the objective pole-piece gap. We investigate the Au-assisted GaAs nanowire growth on silicon-based microelectromechanical systems (MEMS) designed for resistive heating in a reactive environment at the tip of a TEM holder.^27^ TEM holders, designed by Hitachi High Tech Canada, with electrical contacts for MEMS and double tilt capabilities are used. Further details and capabilities of the instrumentation have been reported elsewhere.^28^

**Figure 1:**
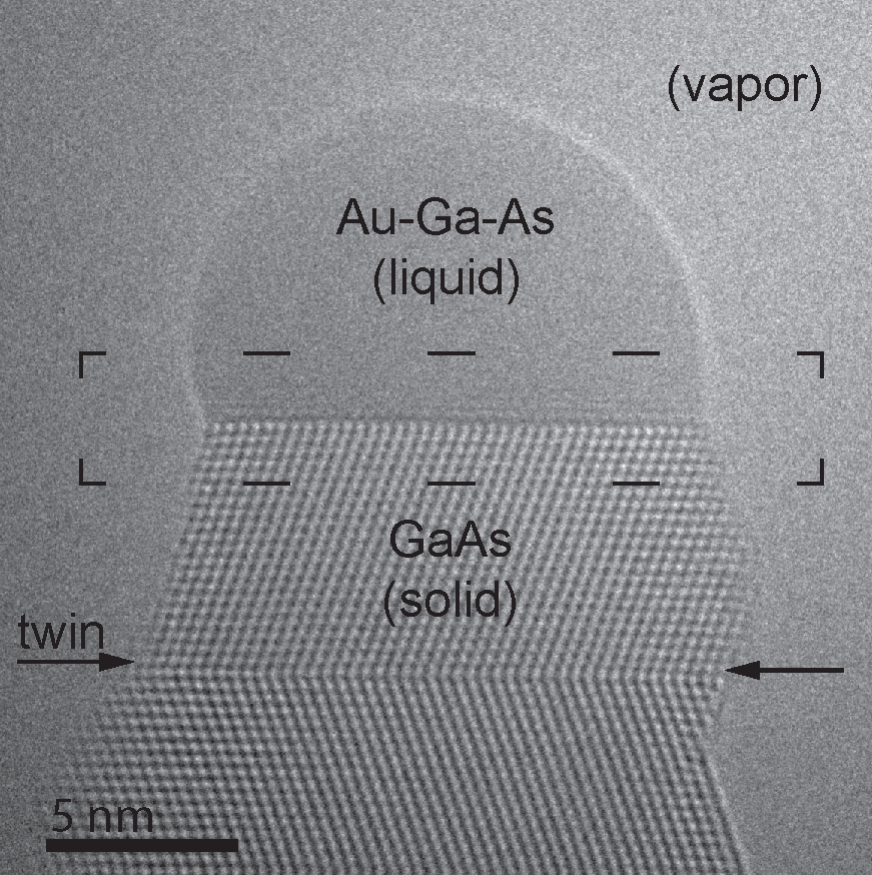
GaAs crystal with a Au–Ga droplet to assist the growth at the crystal–droplet interface that is suited for atomic resolution electron microscopy. The viewing direction ([11̅0]) allows us to distinguish the atomic stacking sequence being able to separate normal stacking from twinned (arrow). The dashed rectangle highlights the liquid–solid growth interface which is here investigated in detail with respect to formation and propagation of individual crystal layers. Details on the crystal and its defects, including Fourier transform of the crystal regions of interest, are provided in the Supporting Information (section S1).

We study the formation of Au-assisted GaAs crystals with a twinned zinc blende crystal structure, as illustrated in Figure 1, at average growth rates of 0.2–1 bilayers/s for nanowires with growth interface diameters between 12 and 32 nm. The desired structure and growth rates are achieved by a continuous supply of trimethyl-gallium (TMGa) and arsine (AsH_3_) to the sample within the TEM, at constant flows in ranges of 8.9–44.6 μmol/min of AsH_3_ and 0.006–0.24 μmol/min of TMGa depending on the diameter of the growing interface. The experiments of this study are conducted at a target temperature of 420 ^◦^C set by resistive heating and total column pressure ranging from 0.2 to 1.7 Pa, depending on the gas flow.

We continuously image the growth of GaAs in the [11̅0] viewing direction using conventional, *i.e.*, using parallel illumination, TEM and (video) record the process using a Gatan OneView IS system with exposure times of 10–100 ms. The recordings are then used to evaluate the growth process, both the time needed for material buildup in the liquid prior to formation of a 2D island (incubation) and the time for the propagation to complete the layer. For comparability of different structures, we investigate both the normal (untwinned) crystal growth leading up to a twin forming as well as the twin formation itself. This provides an internal reference for each nanowire at the specific gas flow and nanowire diameter.

The acquired data of twinned and normal crystal layers are used to develop a thermodynamic model demonstrating when and how twins are formed and elaborating on how they differ from regular growth.

## Results and Discussion

**Figure 2:**
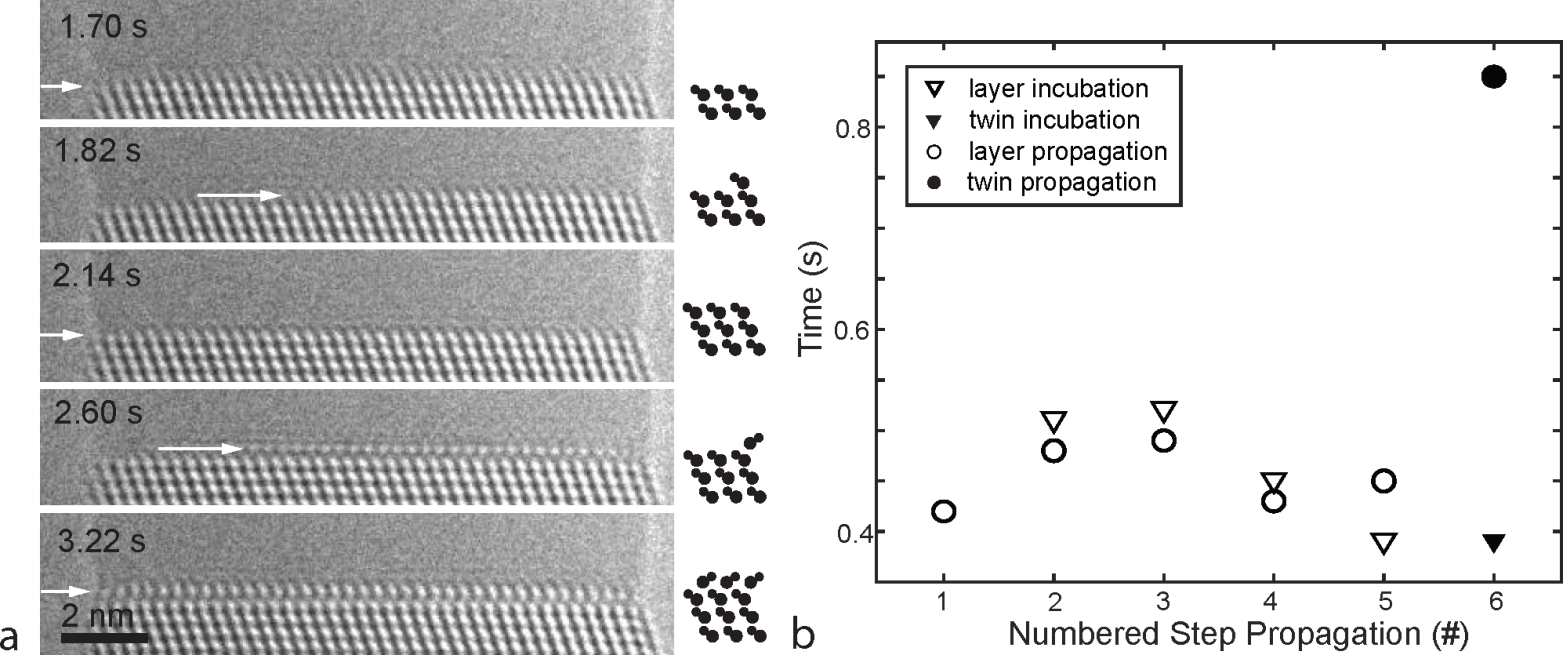
Image series (a) showing the sequential propagation of a normal step and that of a twin, along with illustrations of the evolving atomic stacking. The arrows act as a guide to follow the propagating step. The image series is provided in video format in the Supporting Information (Movie S1) and is part of the data set presented in (b). The plot shows the incubation (triangles) that precedes the nucleation and the time needed to propagate the layer (circles), both for the normal crystal layers (open) and twinned (filled).

From the microscopy growth studies, we observed layer-by-layer formation of both normal stacked bilayers (with respect to the underlying crystal) and those of twinned stacking configuration, as shown in the sample image sequence in Figure 2a. In this sequence (shown for a nanowire with an interface diameter of 12 nm), the top image shows the nanowire between layer growths (no growth occurring), while the next two images show two instances during the propagation of a normal crystal layer. The fourth and fifth images in the series show the propagation of a new layer which is twinned relative to the underlying crystal layers. The figure depicts the critical ability to distinguish the atomic layer configurations during the propagation itself. We observe that the new layers typically start in contact to the edge of the nanostructure, as indicated by 1.82 s into the image sequence in Figure 2a or in the corresponding recording provided in the Supporting Information (Movie S1). This observation is similar to those of current reports on the formation of droplet-assisted GaAs growth in the hexagonal phase,^3^ thus highlighting similarities between the formation of different crystal structures. The 6-fold symmetry of the structures means however that some nucleation events will occur at edges appearing toward the middle of the structure in the projection. Since the viewing direction of [11̅0] is required to distinguish the stacking position of the crystal layers, it is therefore not possible to unambiguously determine the nucleation position of each layer; however, that is not necessary for this study.

The measured growth dynamics for this nanowire are plotted and presented in Figure 2b, with triangles indicating incubation (the time between the completion of the previous layer and the initiation of the new layer) and with circles for propagation (the time required for the layer to finish growing). Here, the first five layers (open symbols) grow in a normal configuration, while the last presented layer (filled symbols) is twinned relative to the underlying crystal. The data set shows two notable aspects of twin formation. First, we note that the incubation times (triangles) between each layer forming are similar for the twinned layer as for the normal layers (within the variation of the data set). This suggests that the nucleation barrier for a twinned nucleus is similar to that of a nucleus with normal stacking. On the other hand, we note that the propagation of the twinned layer is significantly slower than that of normal configured layers (compare filled to open circles). The longer completion time of a twin agrees to a first approximation with the theoretical works which reports that the cohesive energy of a twin is higher than that of the normal zinc blende structure.^18,29^ A higher cohesive energy of the solid reduces the supersaturation of the liquid with respect to the crystal, which corresponds to a reduced rate of crystallization.

**Figure 3:**
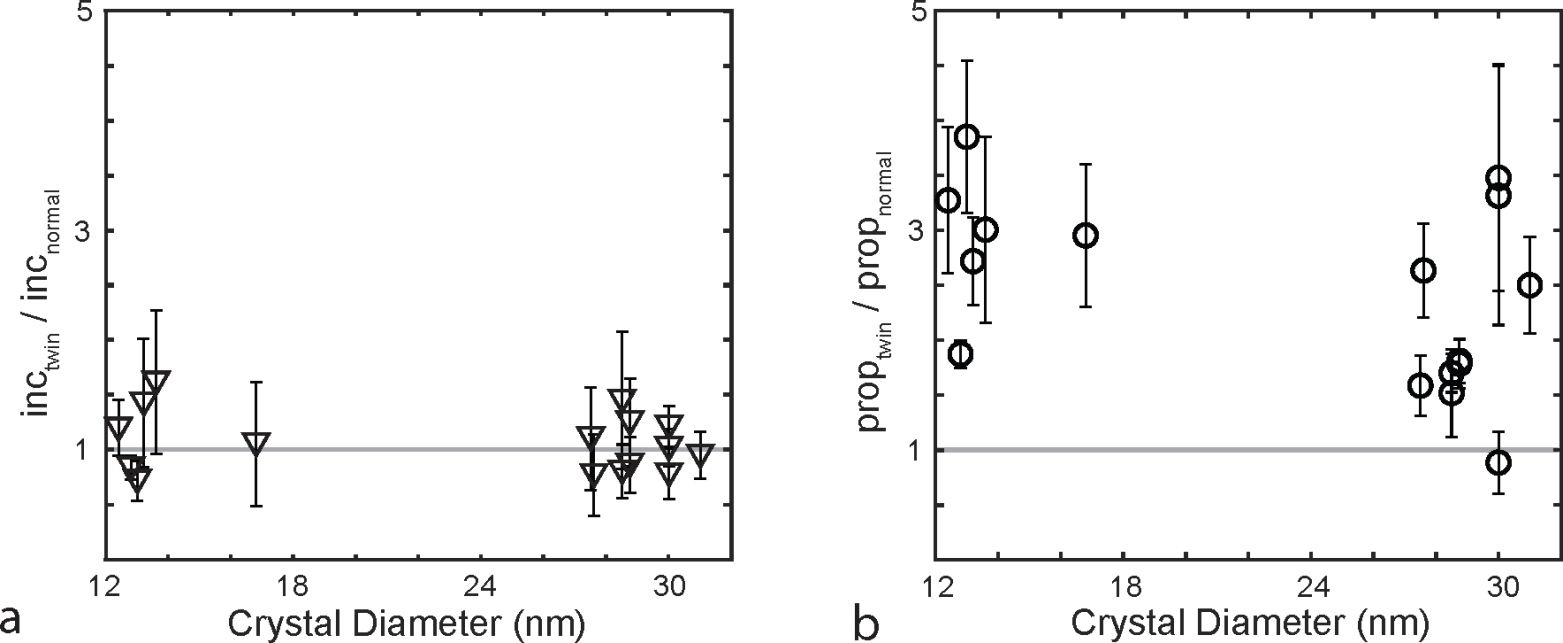
Incubation (a) and bilayer propagation (b) when comparing the twin formation to the normal stacking sequence. The solid line at ratio 1 acts as a guide for when the properties are identical for the twinned and the normal case. This data set is also provided in the Supporting Information (section S2) with superimposed information on the number of layers formed with the twin.

We next consider the growth dynamics of a larger set of nanowires with varied diameter and different growth conditions to determine the generality of the observations. Incubation times and layer propagation times are collectively shown in Figure 3a and b, respectively. Here, the characteristic times for a twin layer are shown relative to the average of the values for, at least, the preceding three layers grown in a normal configuration. We can see in Figure 3a that across the set the incubation time for the twinned layer occurs as fast as the average incubation time for normal layers (where the gray line indicates a ratio of twin/normal of 1). On the contrary, we see in Figure 3b that the propagation time of a twinned layer is almost exclusively longer than the average for the preceding normal layers over the entire diameter range studied (often with a factor of 2 or higher). Further individual data sets are presented in the Supporting Information (section S3) for crystals of 30 nm diameter

In order to understand the observations above, we next consider the thermodynamics of the layer formation. The growth conditions used here to form the twinned zinc blende structure yield relatively slow step propagation, when compared to similar *in situ* studies of Au-assisted GaAs.^20^ This indicates that the supersaturation of the droplet with respect to a zinc blende stacking sequence is rather low.^30,31^ With the added information that the twinned layer propagates more slowly than the normal layer, we get insight into the energy gain for building the step in the respective cases. Together with the observation of similar incubation times, which implies comparable nucleation rates between the configurations, we can formulate a thermodynamic description that qualitatively describes the experimental observations.

**Figure 4:**
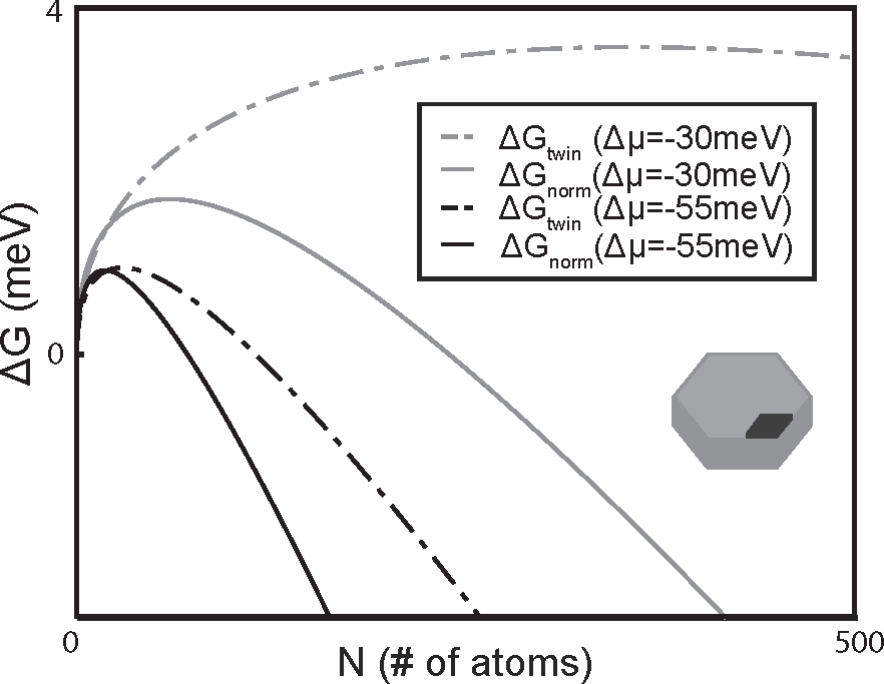
Illustrative graph showing the qualitative behavior of the change in Gibbs free energy for zinc blende formation at low supersaturation of the droplet for formation of a twin (dashed line) or nontwin (solid line), starting from a rhombic island at the triple-phase line (see schematic inset). Reducing the supersaturation from, in this example, −55 meV/pair (black) to −30 meV/pair (gray) gradually increases the nucleation barrier and lowers the energy gain for adding atoms to a stable cluster. The full range is provided in the Supporting Information (section S4). (σ_iL,zb_ = 0.3 J/m^2^;^30^ σ_iL,twin_ = 0.4σ_iL,zb_;^36^ σ_iV_ = 1.5 J/m^2^;^37^ Δγ_zb_ = 0 J/m^2^; Δγ_twin_ = 0.024J/m^2^;^18^ β = 105°).

Nucleation of a new layer during VLS growth is typically considered to take the form of a 2D island. There is an energy cost to forming this island due to the formation of new edges (toward the vapor and toward the droplet [liquid]). An island bonded in a hexagonal (twinned) configuration is typically considered to have lower edge energies than an island with cubic configuration, but is also associated with a relatively higher cohesive energy. This can result in similar nucleation barriers for cubic and hexagonal configurations (normal and twinned layers, in this context), enabling structural mixing and wurtzite phase.^29,32^ We can address the formation of twinned layers using a similar classical nucleation approach to those used to describe formation of the new metastable wurtzite phase or zinc blende/wurtzite interfaces.^30,33^ The energy difference between twin and normal layers has been calculated from a binding energy perspective using approximation of nearest-neighbor interaction or generalized gradient approximation and reported in literature.^18,29^ The change of Gibbs free energy (Δ*G*) for forming a twin layer can be written as
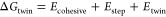
1This takes into account the energetic gain of cohesive energy (*E*_cohesive_) for the solidification of zinc blende (zb), governed by the difference in chemical potential (Δμ; supersaturation). This is balanced against the contribution from step energy (*E*_step_) due to the line energies (σ_*xy*_, where *x* and *y* are the phases that forms the interface) added by forming an island atop a planar surface scaled by the length of each edge (*l*_*iV*_, *l*_*iL*_). Formation of a twin layer involves an additional energy term (*E*_twin_) due to the creation of new interface (Δγ_*j*_) between the twinned island, the droplet, and the underlying crystal. The three terms can then be expanded as follows to be applicable to the stacking configuration *j*:
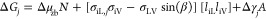
2where *N* is the number of atoms incorporated into the growing layer, creating the interface area *A*. The line energy terms (σ) define the energies of forming steps at interfaces separating the island (i), liquid (L), or vapor (V) from each other with the conventionally defined droplet wetting angle (β).^34,35^ This expression is illustrated in Figure 4 in which the formation of a normal and twinned layer is compared (solid and dashed lines, respectively) for two cases of different supersaturation (higher supersaturation, black; lower supersaturation, gray). The range of supersaturation used here are intentionally bounded by reported Monte Carlo simulations of GaAs zinc blende formation at low supersaturations (>-55 meV/atom pair).^30^

If we first look at the case of the higher supersaturation presented (−55 meV), black lines of Figure 4, we see that the change in free energy first increases with the size of the nucleus/growing layer and then decreases. This nucleation barrier is similar for the normal and twin layer at the relatively high supersaturation (compare solid and dashed black lines). Further, the twinned layer theoretically requires a larger critical nucleus to form (the cluster size at the nucleation barrier), which would imply a slower nucleation rate than that of a normal layer. Since nucleation rate is tied to the probability of competing configurations,^30^ this agrees with our observations of observed higher nucleation probability of the nontwinned configuration in comparison to the twinned (that is, relatively few of the grown layers have the twin configuration). However, the difference in time scale of the nucleation rates is beyond the temporal resolution limit of our experimental setup (0.01 ms) as we are unable to see difference in incubation (Figure 3a), but the experimentally observed frequency of twin formation provides the subtle statistical difference in nucleation probability. We can further see in Figure 4 that the energy gained for adding an atom to a stable cluster (∂Δ*G*/*∂N*) is significantly lower for the twinned case at the same supersaturation of the droplet, as seen by the flatter slope beyond the nucleation barrier. In other words, continuous growth of a twinned layer (black dashed line) theoretically proceeds slower than a normal configured layer (black solid line), in line with the experimental observations presented in Figure 3. While this analysis is presented for a static supersaturation, the characteristics of the two cases presented in combination with the presented *in situ* data provides insight into the thermodynamic process and additional energy requirement for forming a twinned bilayer. The experimental observations of twin formation can be replicated with an additional energy of approximately 20 mJ/m^2^, which is in line with the calculated value for the collective bond energies of a twin plane (24 mJ/m^2^).^18,29^

During VLS growth, nucleation of an island results in a reduction of Δμ,^24,38^ such that layer propagation occurs under much lower supersaturation conditions than the nucleation. This in turn leads to a significant change in Gibbs free energy when comparing the two different stacking configurations, shown as gray in Figure 4. Especially, the model predicts the twinned energy landscape to be significantly flattened by the reduction of Δμ, which corresponds to a reduced energy gain for adding atoms to the stable twinned cluster. While the same thing can be said for a normal configured layer (solid line), the change is not nearly as drastic as for the twinned layer (dashed line). Reducing the ∂Δ*G*/*∂N* translates to a reduction in propagation rate of the formed island and would therefore theoretically result in an extremely slow process of propagating a twin, when compared to propagation of a normal layer. Moreover, it is clear that this will be especially true when the crystal growth is already occurring for relatively low supersaturation.

**Figure 5:**
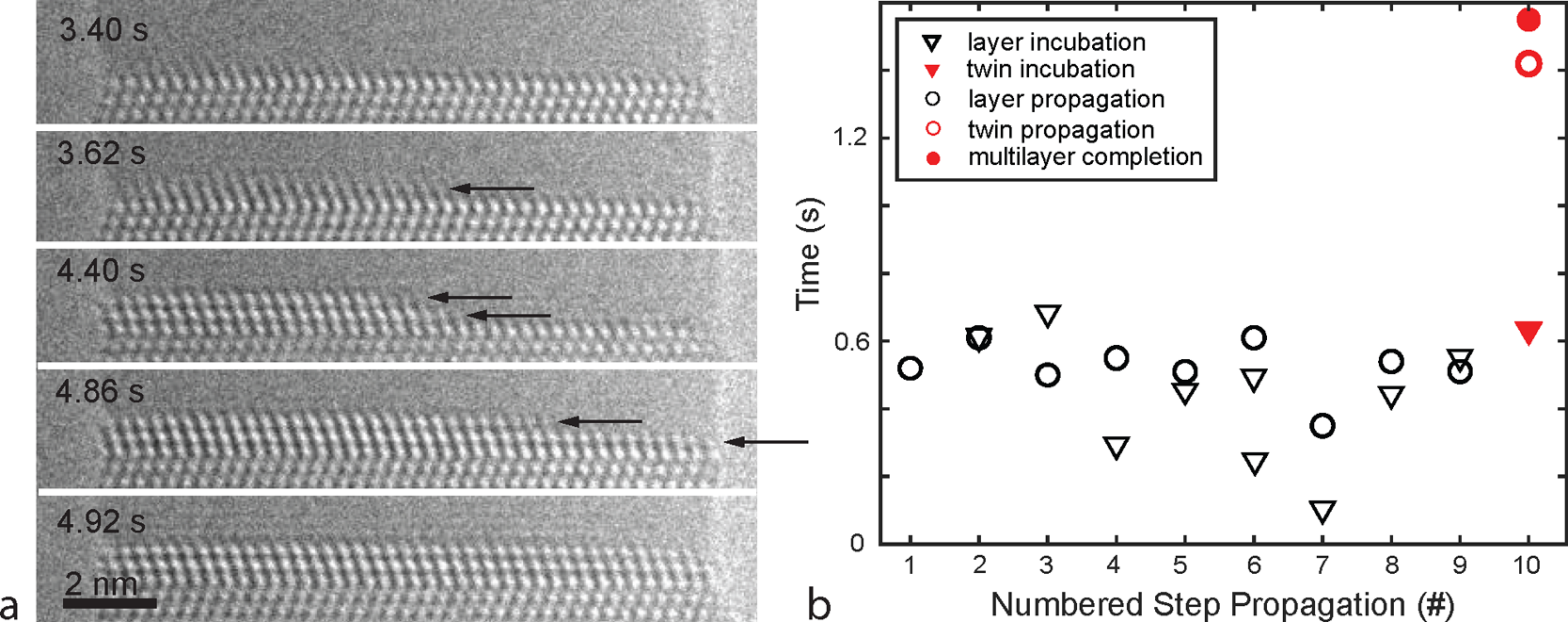
Image series (a) of the formation of a twin with one additional layer and the corresponding data set (b) of incubation and step propagation times leading up to it. We separate the completion of the twin and the multilayer segment as they are propagating simultaneously as seen at 4.40 and 4.86 s into the image series, resulting in two propagation times for the twinned case, with one marking the completion of the twinned layer (open red circle) and one corresponding to completion of both layers in the multilayer stack (closed red circle). The image series is provided in video format for the reader in the Supporting Information (Movie S2).

For some cases of twin formation, we observe that additional layers can be formed and propagate on top of the twin, even before the twin has propagated to completion. In these cases, several layers propagate simultaneously. This is shown as an image series of the growth interface in Figure 5a, where the twinned layer is observed to form and propagate across the interface (second image in the series) and a second layer in the normal configuration nucleates and grows on top of the twinned layer (third image in the series). The propagation of the additional layer catches up to the growth front of the twinned layer and forms an additional step that propagates along with the twin; see 4.40 s in Figure 5a or the video recording provided in the Supporting Information (Movie S2). Following the propagation, we observe that the completion of the additional layer is slightly staggered from that of the twin (fourth image); however, the completion of the additional layer occurs rapidly after twin completion suggesting it is limited by the twin propagation.

The incubation and propagation are plotted separately and shown in Figure 5b, where black symbols indicate incubation and propagation of layers prior to the twin while red symbols indicate the twin layer. As in the case of a single layer twin, we observe the twin layer forming with similar incubation as the normal crystal layers but with slower propagation. We distinguish between the propagation time of the individual twin layer (open red circle) and the time to complete the entire multilayer segment (filled red circle); these are of course inherently connected since the two simultaneously growing layers compete for material from the droplet. We note that the additional layer formed on top of the twin is of a regular stacking with respect to the twin, and as such would be assumed to follow the thermodynamics of a nontwinned layer.

This multiple layer formation is observed, and suggested to occur, at the slow step propagation of the twin formation. In the case of binary crystal growth with depletion of either of the growth components following nucleation, we would generally assume the supersaturation of the droplet to remain low until the layer has been completed.^20,34^ However, since we observe additional nucleation events, we conclude that the supersaturation of the droplet must build up during the relatively slow propagation of the twin layer in comparison to the normal layer. During supersaturation buildup (*e.g.*, going from −30 meV/pair toward −55 meV/pair) as a result of an overall slow propagation, our experimental observations suggest that the nucleation of a normal configured layer would become increasingly more probable, eventually becoming a competing event with propagation of the twin layer. This situation is presented in its extreme by the case shown in gray in Figure 4; for relatively low overall supersaturation, where the change in Gibbs free energy relative to the growing twinned layer is relatively constant (dashed gray line) and can exceed the barrier for nucleation of a normal layer (solid gray line). In addition, the observation of a new layer forming on top of a propagating twin reveals that the propagation is not restricted by the availability of growth material in this case. Thus, we suggest that the occurrence of additional layers on top of the propagating twinned layer is a result of the limited energy gain of adding atoms to the twinned island, which together with a progressively increasing supersaturation increases the probability for nucleation of an additional layer.

**Figure 6:**
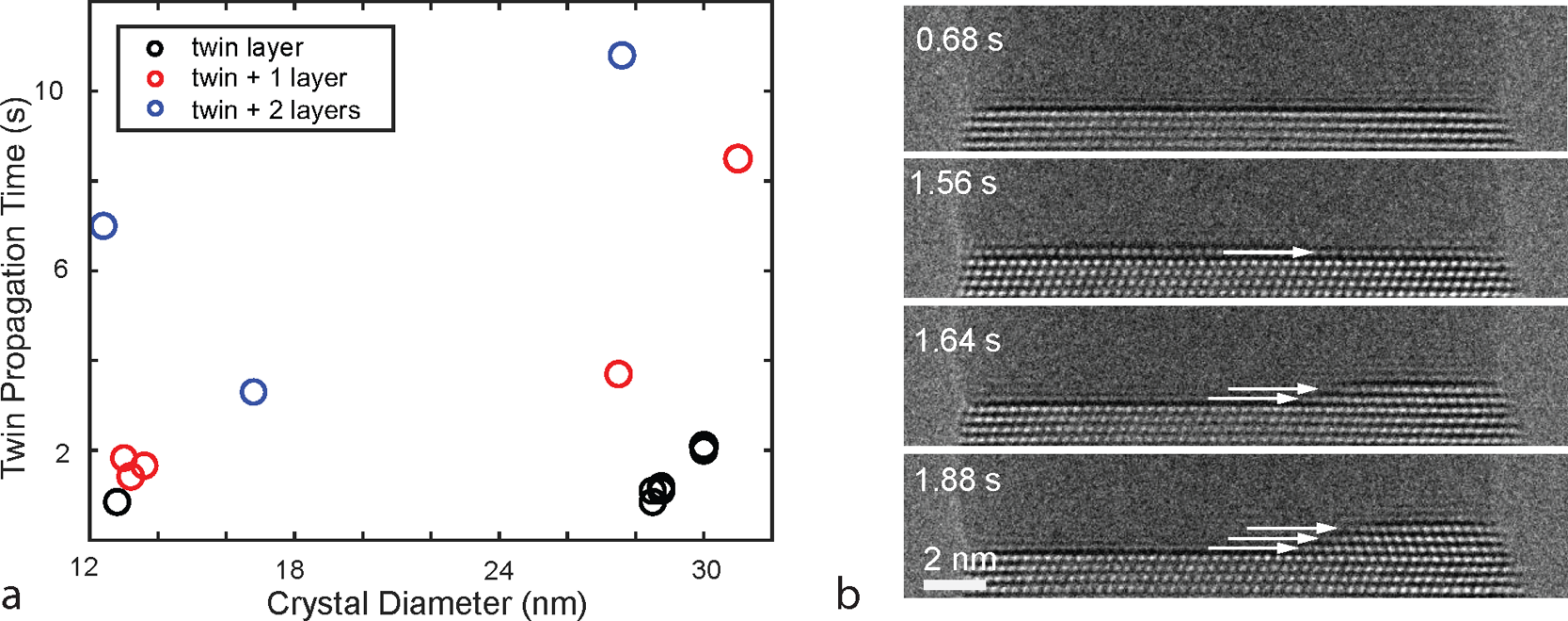
Summary of twin propagation times for all studied nanowire diameters (a) where zero (black), one (red), or two (blue) layers are observed in addition to the twinned layer. The formation of a multilayer segment is presented in the image series (b) showing the formation of a twin and sequentially added normal islands on top of the twinned layer prior to completion of the twinned layer. The image series is presented in video format in the Supporting Information (Movie S3).

This explanation is supported by the fact that we have observed the formation of two additional layers on top of the twin itself at even slower twin propagation as in the case of Figure 6a. The graph shows the collected data with nanowires of a range of diameters (between 12 and 32 nm) for cases where only the twin is formed as a single layer (black) and when it is accompanied by another one (red) or two (blue) additional normal layers. From the data set, multilayer formation is observed at increasingly slower propagation of the twinned step when comparing crystals of similar sizes. While the layer propagation for a single nanowire is governed by the AsH_3_ partial pressure,^20^ we found that the AsH_3_ partial pressure at the sample is not the significant factor for globally controlling the twin propagation time (Supporting Information, section S5). The lack of AsH_3_ dependence on the step propagation is likely a result of conducting growth using a range of crystal growth parameters such as crystal diameters. Being able to separate the arsine pressure from the step propagation strengthens our suggestion that the multilayer formation is enabled by slow twin propagation, which has yet to be reported for growth of a nondefected droplet-assisted nanowire crystal.

The observation of multiple layers propagating at once shows that the layers are formed sequentially and not as a single 3D island. Figure 6b shows how the propagating stack of layers is formed one by one as time progresses and material is supplied to the crystal. The figure shows individual layers being added on top sequentially, and it proceeds to complete the layers whose propagation is strictly limited by that of the twinned layer in the bottom of the stack. In this case, we suggest that a slower twin propagation allows for a faster buildup of Δμ which in turn promotes additional nucleation events in parallel with propagation of the twinned layer. A second and third layer formed in this fashion has always been observed to be of a normal configuration, thus following the normal cubic stacking with respect to the twin.

### Applicability

This observation of multilayer formation during VLS growth challenges the idea that the crystal, in this case nanowire, grows one bilayer at a time. Although the crystal growth is studied under an electron beam, we do not see an effect of the electron dose on the resulting twin formation with regard to multilayer propagation (provided in Supporting Information, section S6) for the doses used here. This implies that the multilayer formation is also probable during conventional crystal growth (without the electron beam). This indicates that multilayer growth is an inherent feature of the layer-by-layer growth when stacking defects and changes of crystal phase occur and thus needs to be considered to achieve true atomic-layer control. Currently, the theoretical approaches to nanowire crystal growth are mainly focused on the assumption that it is limited by the nucleation.^39−41^ However, this assumption is not strictly true for defect formations such as twin layers according to our observations of twinned GaAs, which is the most common compound system for modeling nanowire growth.^30,34,42^

Similar multilayer formation might be anticipated in other cases where the energy landscape is changed during crystal growth. This would include compositional heterostructures, where the material is changed at the interface rather than only in the crystal phase, which are an essential tool in semiconductor device engineering. Nanowire structures grown by VLS have an enormous advantage in heterostructure formation, since lateral strain relaxation greatly reduces the demand for interfacial lattice matching. However, the interface formation dynamics may also be expected to yield multilayer growth for at least some conditions, complicating the formation of atomically precise heterostructure layers. As such, incomplete segment formation at lower supersaturation for VLS-grown InP segments in InAs nanowires may be a consequence of this effect.^43^ However, if the formation of multilayer segments can be understood, it may open new opportunities, for instance, to intentionally embed 3D islands within nanostructures of another material.^44^

## Conclusion

Using direct observation of atomic bilayer formation in Au-assisted GaAs nanowire growth by *in situ* TEM, we investigate the difference in growth dynamics between individual layers with normal (zinc blende) and twinned configuration. For conditions in which twinned layers occur regularly, but less often than normal layers, we show that the incubation time for the nucleation of a twin is comparable to that of a normal layer. In addition, we observe that the propagation (subsequent growth) of the twinned layer occurs more slowly than that of the normal layers. Using a nucleation model for the crystal growth, we show that while the energy barrier to nucleating a twinned layer is similar to that of a normal layer, the energy cost of forming a twinned interface limits the propagation time of the layer. With slower layer propagation, and thus a faster buildup of supersaturation, we observed a higher probability of nucleating additional layers on top of the newly formed twin prior to completing the twinned layer. These observations and conclusions provide new insights to the growth process of defect formation in crystal growth, such as twinned zinc blende crystal structure. This is a step toward detailed understanding for deterministic crystal growth, by gaining knowledge of the formation process when the atomic stacking sequence is altered.

## Supporting Information Available

Fourier transform showing the crystal symmetry around a twin; relative growth of twins with respect to the normal crystal depending on how many additional layers that propagates along the twin; incubation and propagation data for twins formed in nanowires of 30 nm diameter; model representation for cases of intermediate supersaturation; AsH_3_ partial pressure and its influence on the propagation rate; and acquired data correlated with electron dose (PDF)
Crystal growth of twinned zinc blende GaAs (single layer), corresponding to the data sets of Figure 2 (MP4)
Crystal growth of twinned zinc blende GaAs (double layer), corresponding to the data sets of Figure 5 (MP4)
Crystal growth of twinned zinc blende GaAs (triple layer), corresponding to the data sets of Figure 6b (MP4)
