# Supplementary material for: Direct Observations of Twin Formation Dynamics in Binary Semiconductors
Source: ACS Nanosci Au. 2021 Nov 4;2(1):49–56. doi: 10.1021/acsnanoscienceau.1c00021 (PMC10125175; doi:10.1021/acsnanoscienceau.1c00021)
Supplement: Supplementary file 1 — ng1c00021_si_001.pdf [file ng1c00021_si_001.pdf]

Supporting Information: Direct observations of twin formation dynamics in binary semiconductors

Marcus Tornberg,[*,†,‡] Robin Sjökvist,[†,‡] Krishna Kumar,[†,‡] Christopher R. Andersen,[†,‡,¶] Carina B. Maliakkal,[†,‡] Daniel Jacobsson,[†,‡,§] and Kimberly A. Dick[†,‡,§]

†*Centre for Analysis and Synthesis, Lund University, Box 118, 22100, Lund, Sweden*

‡*NanoLund, Lund University, 22100, Lund, Sweden*

¶*National Centre for Nano Fabrication and Characterization, Technical University of Denmark, 2800 Kongens Lyngby, Denmark*

§*National Center for High Resolution Electron Microscopy (nCHREM), Lund University, 22100, Lund, Sweden*

E-mail: marcus.tornberg@chem.lu.se

S1 Symmetry of the GaAs crystal around the twin

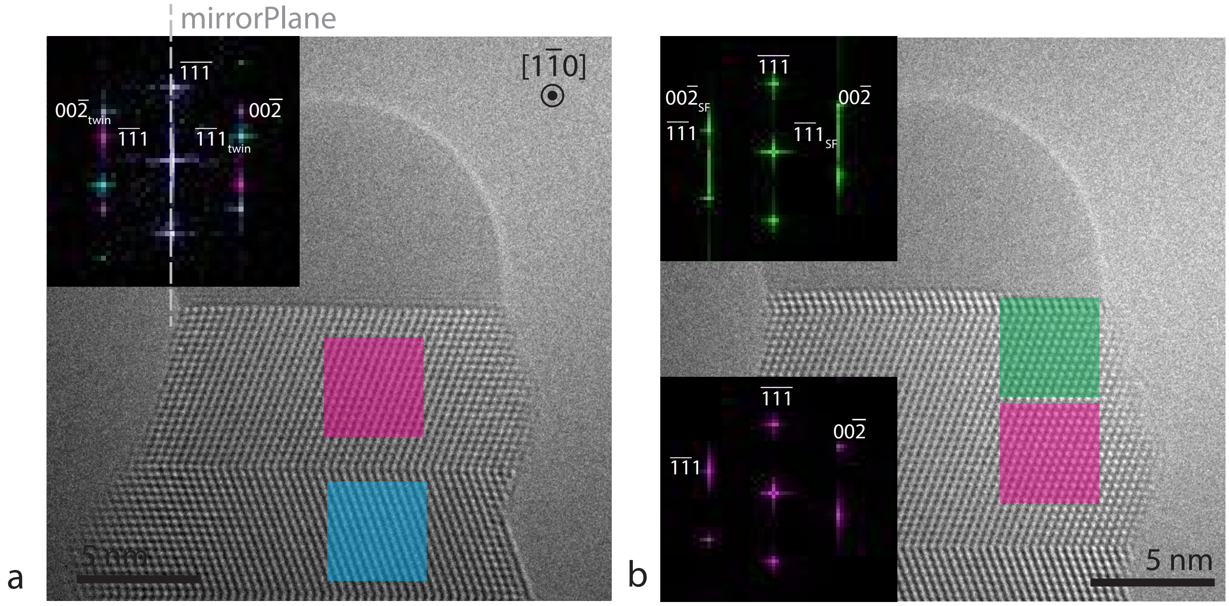

Figure S1: Selected area Fourier transform of both twins shows the orientation and the rotation axis ([111]) of the rotation defect. (a) shows the crystal symmetry of the overview image presented in figure 1 in the main text, showing the Fourier transform of the lower (cyan) and the upper crystal (magenta). The frequency map is labeled for both crystal orientations where the upper crystal is denoted as the twinned version of the lower one.(b) illustrates the fourier transform the "regular" zinc blende (magenta) and the change of the periodicity of the forming stacking fault(green). The additional reflections/streaks within the frequency map containing the stacking fault (SF) matches the expected reflections of the twin. The transforms provides the information of the viewing direction ($[1\,\bar{1}\,0]$) along with the growth direction ($[\bar{1}\,\bar{1}\,\bar{1}]$).

In practice, we rely on the high resolution conventional TEM images to evaluate the change in stacking sequence along the $[\bar{1}\,\bar{1}\,\bar{1}]$-axis. To support this, we provide Fourier transforms of the structure to showcase the crystal symmetry of homogeneous regions of each side of the twin (figure S1a). It shows that the rotation caused by the twin formation is revolving around the $[\bar{1}\,\bar{1}\,\bar{1}]$ axis. The inset of the panel shows the superimposed transforms of both regions, compare the magenta and cyan in the superimposed pattern in the inset. This can be extended to newly formed twin layers by utilizing the Fourier transform from a homogeneous crystal region (magenta of figure S1b). By then transforming an area containing the first formed layer (green) we observe occurrence of additional reflections showing as streaks in addition to the expected pattern of a pure crystal (magenta). The reflections shows as lines as we are transforming the one dimensional projection to a frequency map. Since the object size in frequency space is inversely proportional to its real space size, lines will show rather than spots in the direction perpendicular to the defected plane. The additional "reflections" matches the reflections that would stem from a larger area of a twinned region with respect

to the previous crystal, compare to the cyan data of figure S1a.

S2 Comparing twin and normal growth dynamics

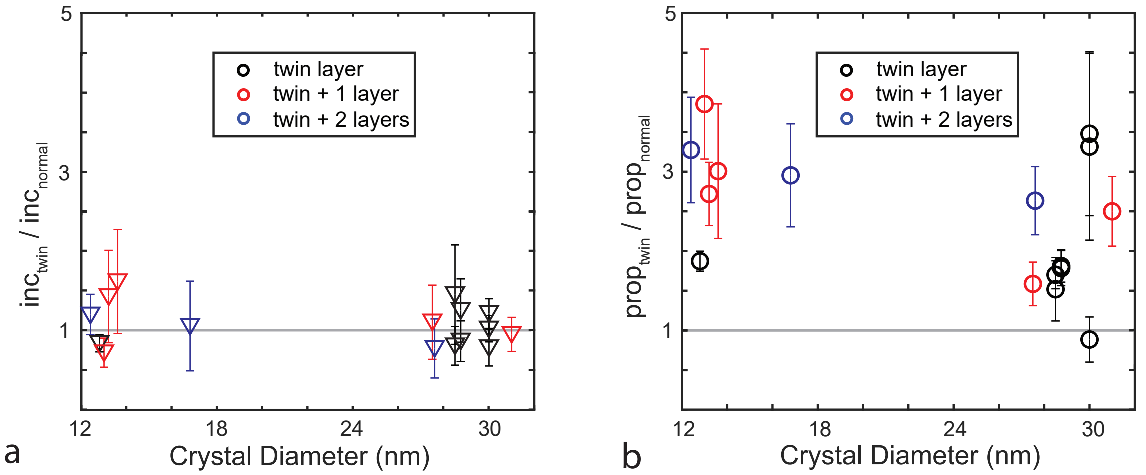

Figure S2: Here we present figure 3 from the main article with the added information of how many layers that were formed in conjunction with the twin.

Figure 3 presented in the main article is a collection of the twins that we have studied, including isolated twins, and those that are associated with multilayer growth. Figure S2 superimposes data for when zero, one or two additional layers have been formed as a result of a twin forming. Interesting to note, there is no particular correlation between the ratio presented and the resulting twin formation (multilayer or not). This further supports the notion that it is the actual propagation time of the twin rather than the relative propagation time that influences the growth of multiple layers simultaneously.

S3 Growth Dynamics for larger growth interfaces (30 nm)

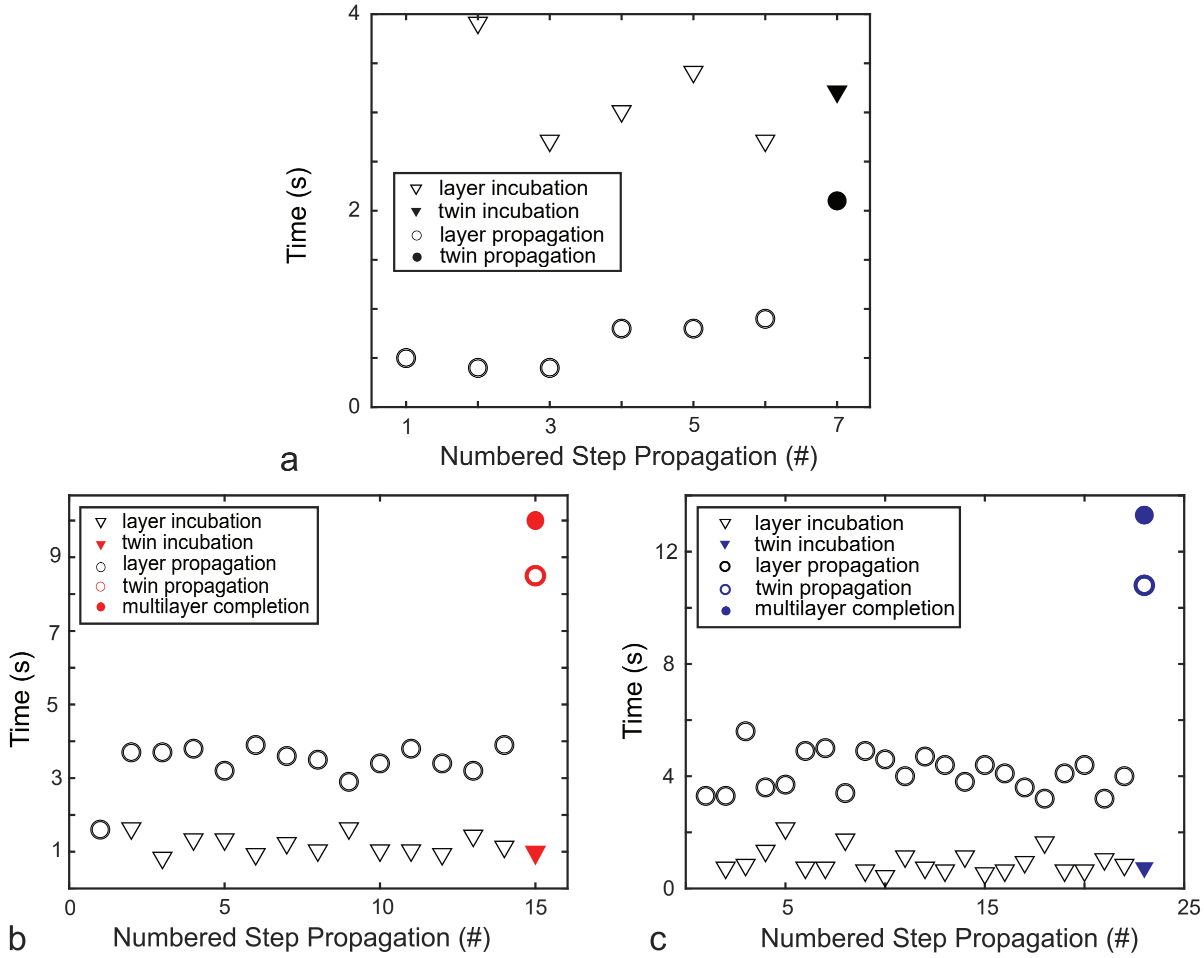

Figure S3: Three individual datasets for incubation and propagation of normal and twinned layers shown for isolated twins (black), and in the cases where the multiple layers are propagating on top of the twin. (red – 1 additional layer, blue – 2 additional layers). The datasets presented here are collected from nanowires with diameters of 30.0 nm (a), 31.0 nm (b) and 27.6 nm (c). Note that even though the growth rate varies from layer to layer, the twin layer formation is still distinct from the normal growth.

For transparency, we provide individual *in-situ* datasets from three different nanowires of greater diameters than presented in the figure 2 and 5 of the main article. Figure S3 shows the layer by layer growth leading up to the twin (open black data) for cases of an isolated twin layer (filled black data) and in the cases where one (red) or two (blue) additional layers are nucleated prior to the completion of the twinned layer. While the datasets present data from similar crystal diameters they are part of separate experiments where this has been observed.

S4 Model at intermediate supersaturations

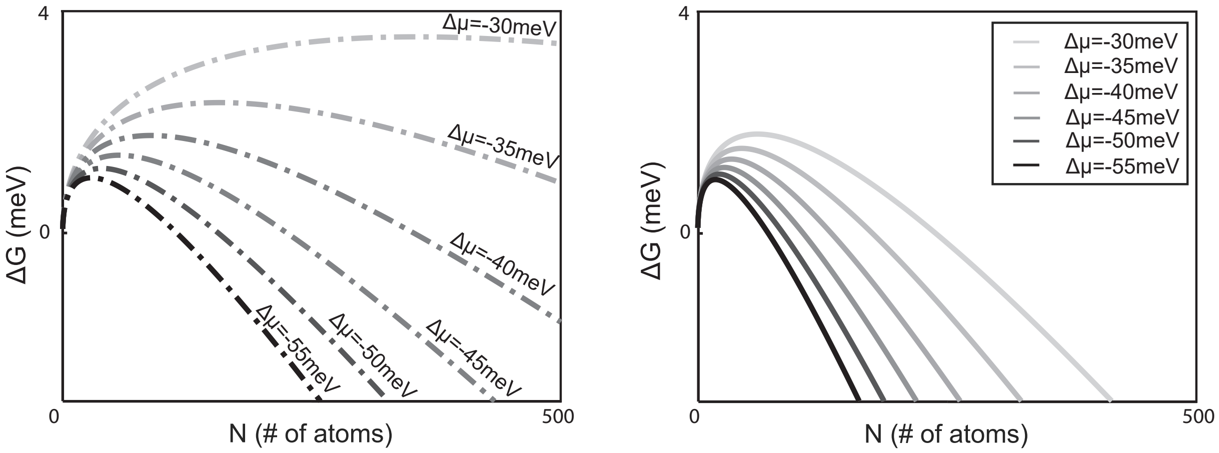

Figure S4: Presenting the calculated change in Gibbs free energy for intermediate values with respect to those showcased in the article figure gibbs. It is shown for both twinned (dashed lines) and normal, untwinned, layers (solid lines) at the same scale for both panels.

For clarity, we present the change of Gibbs free energy for supersaturation which are between the extreme cases presented in the main text (-55 meV/pair and -30 meV/pair). Figure S4 shows the behavior of the energy as the supersaturation changes, showing that the twinned case (dashed lines) is more affected by successively lowering the supersaturation ($\Delta\mu$) of the droplet with respect to the zincblende crystal than in the case of a normal zincblende layer (solid lines).

S5 Influence of Arsine on the propagation of twin layers

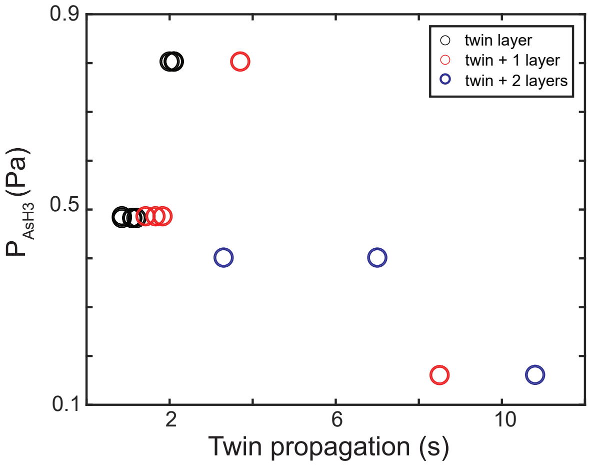

Figure S5: The pressures of AsH_3 and its relation to the propagation of the twinned layer. This illustrates the range of partial pressures of AsH_3 and showcases that it is not directly related to the twin propagation. In addition to the relationship, the number of normal layers that propagates in parallel with the twin is presented in color (black – 0, red – 1 and blue – 2).

With the reported correlation between arsine partial pressure and step propagation during normal growth in mind, we evaluated the impact of the partial pressure of the experiments presented in the main article on the twin propagation as shown in figure S5. In our study, we do not observe the same correlation when investigating the twin propagation, likely due to studying a larger spread in diameter across our experiment. Instead, this highlights that the observation of multiple layer formation along with twins is likely related to the propagation rate rather than the AsH_3 partial pressure.

S6 (Lack of) electron beam effect on mutlilayer formation

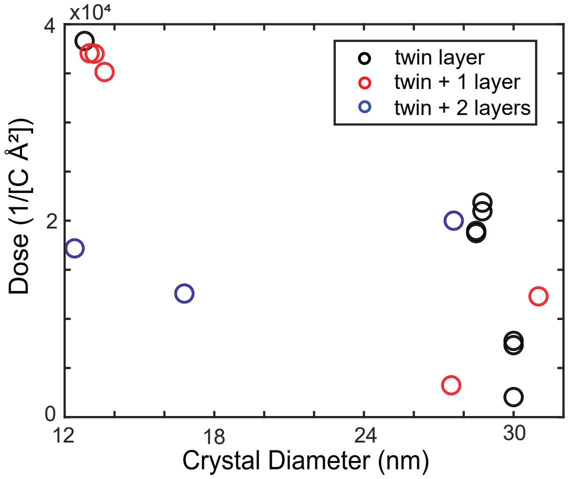

Figure S6: The electron dose as a function of crystal diameter shows that the twinned layer (as part of a mulitlayer or not) is not determined by the exposure. We see twins with and without additional nucleated layers over the full span of electron doses used for imaging.

The fact that this study uses *in-situ* microscopy raises the question of the beam influence of the observations. Following that we present the twin propagation measurements and the crystal diameter vs the measured dose at the time of twin formation. The dose measurement is made from vacuum areas surrounding the object of interest within the images acquired using the calibrated Gatan OneView IS system. Figure S6 presents the correlation between electron dose (electrons/Å^2) and the crystal diameter, with the multilayer classification as coloration. From our sample size, we are not able to see an effect of the electron exposure on the twin formation with respect to multilayer. An observation which strengthens the observation and its applicability to the general crystal growth (outside of an electron microscope).
